# The Role Played by Novel Inflammatory Markers in Assessment of Peripheral Artery Disease

**DOI:** 10.3390/medicina59091557

**Published:** 2023-08-28

**Authors:** Viviana Onofrei, Adrian Crișan, Cristina Andreea Adam, Dragos Traian Marius Marcu, Mihai Ștefan Cristian Haba, Laura Carina Tribus, Alexandr Ceasovschih, Irina Mihaela Eșanu, Antoneta Dacia Petroaie, Radu Crișan-Dabija, Maria-Magdalena Leon-Constantin, Carmen Cumpăt, Florin Mitu

**Affiliations:** 1 Department of Medical Specialties I and III, “Grigore T. Popa” University of Medicine and Pharmacy, University Street No. 16, 700115 Iasi, Romania; 2 “St. Spiridon” Clinical Emergency Hospital, Independence Boulevard No. 1, 700111 Iasi, Romania; 3 Clinical Rehabilitation Hospital, Cardiovascular Rehabilitation Clinic, Pantelimon Halipa Street No. 14, 700661 Iasi, Romania; 4 Clinical Hospital of Pneumophthisiology Iași, Doctor Iosif Cihac Street no 30, 700115 Iasi, Romania; 5 Department of Internal Medicine, Faculty of Dentistry, “Carol Davila” University of Medicine and Pharmacy, 050474 Bucharest, Romania; 6 Department of Internal Medicine, Ilfov County Emergency Hospital, 022104 Bucharest, Romania; 7 Department of Family Medicine, Preventive Medicine and Interdisciplinary, “Grigore T. Popa” University of Medicine and Pharmacy, University Street No. 16, 700115 Iasi, Romania; 8 Department of Management, “Alexandru Ioan Cuza” University, Blv. Carol I, 700506 Iasi, Romania; 9 Academy of Medical Sciences, 030167 Bucharest, Romania; 10 Academy of Romanian Scientists, 700050 Iasi, Romania

**Keywords:** peripheral artery disease, biomarkers, inflammation, cardiovascular risk, neutrophil-to-lymphocyte ratio, platelet-to-lymphocyte ratio, lymphocyte-to-C-reactive protein ratio

## Abstract

*Background and Objectives*: Atherosclerosis is a multifactorial process in which inflammatory markers have both therapeutic and prognostic roles. Recent studies bring into question the importance of assessing new inflammatory markers in relation to the severity of peripheral artery disease (PAD), such as the neutrophil-to-lymphocyte ratio (NLR), platelet-to-lymphocyte ratio (PLR) and lymphocyte-to-C-reactive protein ratio (LCR). *Materials and Methods*: We conducted a retrospective and descriptive study including 652 patients with PAD, who were divided into two groups according to the severity of the ankle–brachial index value: mild and moderate obstruction (257 patients) and severe obstruction (395 patients). We evaluated demographics, anthropometric data and clinical and paraclinical parameters in relation to the novel inflammatory biomarkers mentioned above. *Results*: Weight (*p* = 0.048), smoking (*p* = 0.033), the number of cardiovascular risk factors (*p* = 0.041), NLR (*p* = 0.037), LCR (*p* = 0.041) and PLR (*p* = 0.019), the presence of gangrene (*p* = 0.001) and the number of lesions detected via peripheral angiography (*p* < 0.001) were statistically significant parameters in our study. For the group of patients with severe obstruction, all three inflammatory biomarkers were statistically significantly correlated with a serum low-density lipoprotein–cholesterol level, the number of cardiovascular risk factors, rest pain, gangrene and a risk of amputation. In addition, directly proportional relationships were found between NLR, PLR and the number of stenotic lesions (*p* = 0.018, *p* = 0.016). Also, NLR (area under the curve <AUC> = 0.682, *p* = 0.010) and PLR (AUC = 0.692, *p* = 0.006) were predictors associated with a high risk of amputation in patients with an ABI < 0.5. *Conclusions*: in our study, we demonstrated the importance of assessing inflammatory markers in relation to the presence of cardiovascular risk factors through the therapeutic and prognostic value demonstrated in PAD.

## 1. Introduction

Peripheral arterial disease (PAD) is one of the main atherosclerotic cardiovascular diseases, and its prevalence has increased, despite the large-scale implementation of primary prevention strategies in recent years [1]. More than 50% of patients with PAD are asymptomatic, leading to a high rate of complications related to increased morbidity and mortality in the absence of multidisciplinary and integrative management approaches used to reduce the risk of a potentially fatal acute vascular event [2,3,4].

In general, 3–4% of amputations that occur annually have obstructive atherosclerotic lesions as their morphopathological substrate, leading to negative prognostic effects in the medium and long term. From a pathophysiological point of view, the atherosclerotic process has a multifactorial origin, with inflammation playing an important role in mediating the processes involved in the progression and destabilization of atherosclerosis [5,6].

PAD is frequently associated with both classic and new cardiovascular risk factors. Of the latter factor type, the pro-inflammatory status, through new inflammatory markers with anti-inflammatory roles, has attracted the interest of the scientific community both in terms of its potential prognostic role and future therapeutic targets among patients with PAD and HF.

The complete blood count is one of the most common biological determinations, and it can provide details associated with the presence of a pro-inflammatory status [2,7]. Recent studies published in the literature have demonstrated the prognostic roles of several inflammatory markers derived from complete blood count and lipid profile analysis performed in these patients. The neutrophil-to-lymphocyte ratio (NLR), platelet-to-lymphocyte ratio (PLR), white blood cells-to-mean platelet volume ratio (MPV), and lymphocyte-to-C-reactive protein ratio (LCR) are potential inflammatory biomarkers with prognostic value among patients with PAD [8]. The systemic immune–inflammation index [9], monocyte-to-high-density lipoprotein (HDL) cholesterol ratio and lymphocyte-to-HDL ratio [10] are biomarkers with important roles in atherogenesis, in addition to those previously mentioned roles.

The possibility that easy and accessible dosing of these markers can provide meaningful clues regarding the patient’s evolution is the main incentive to explore them. The studies available in the literature to date have separately addressed the issue of pro-inflammatory status in relation to PAD or cardiovascular risk in general.

Recent data from the literature bring to light a number of new, easy-to-use and reproducible inflammatory molecules, such as the neutrophil-to-lymphocyte ratio (NLR), platelet-to-lymphocyte ratio (PLR) and lymphocyte-to-C-reactive protein ratio (LCR) [11,12]. Their use as markers associated with the presence of cardiovascular risk factors or severity of obstruction and clinical picture of patients with PAD is limited to date, which justifies the current study. NLR and PLR have previously been shown to be predictors of the risk of an acute vascular event occurring [10,13]. These molecules may also serve as future therapeutic targets for these patients, as the prognostic role of anti-inflammatory medication in patients with atherosclerotic disease has previously been demonstrated in numerous clinical trials [14,15]. Nucleotide oligomerization domain-like receptor family, pyrin domain containing 3 (NLRP3) and interleukin 6 (IL-6) are other inflammatory markers that have a demonstrated role in modulating the inflammatory processes involved in the development and progression of atherosclerosis [16]. In the case of NLRP3, previous clinical research has demonstrated the existence of elevated serum levels of this marker in patients with PAD, together with evidence of a correlation between this marker, macrophage accumulation and the degree of calcification of the arteries [17].

In this study, we aim to identify a series of clinico-biological particularities by analyzing a group of patients diagnosed with PAD, as well as assess the efficiency and efficacy of using new inflammatory biomarkers, and, therefore, provide practicing cardiologists with a feasible and easy-to-apply tool with both prognostic and therapeutic roles.

## 2. Materials and Methods

### 2.1. Study Design

**Figure 1:**
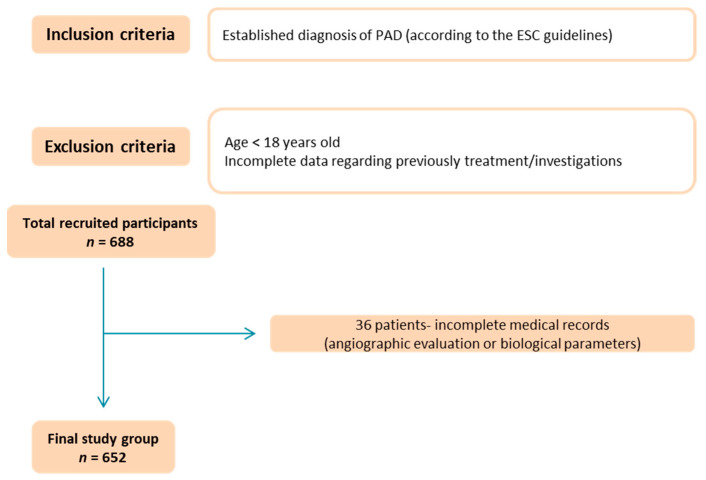
Flow chart of the studied group (PAD: peripheral artery disease; ESC: European Society of Cardiology).

We conducted a retrospective and descriptive study involving 688 consecutive patients diagnosed with PAD and evaluated in the Cardiology Department of “St. Spiridon” Hospital. Thirty-six patients were excluded from the initial group due to incomplete medical records (angiographic evaluation or biological parameters). Thus, the final study group consisted of 652 patients with PAD evaluated in a multidisciplinary manner (Figure 1).

Inclusion criteria were being over 18 years of age, a definite diagnosis of PAD according to the clinical guidelines of the European Society of Cardiology [18] and the provision by those who wished to participate in this study of signed and informed consent. Exclusion criteria were being under 18 years of age, a lack of informed consent and incomplete medical records.

In the absence of a definite diagnosis of PAD established via vascular Doppler ultrasound or peripheral angiography, the presence of symptoms suggestive of PAD was assessed. Symptoms suggestive for PAD were intermittent claudication (IC), presence of paresthesia in the lower limbs, a lack of pilosity, cold and pale skin, petechiae and the presence of dermatitis or ulcers caused by decreased vascularity.

### 2.2. Measurements

#### 2.2.1. Comorbidities and Laboratory Data

We included demographic, anthropometric and paraclinical (biological and imaging) parameters in our study. Anamnesis revealed the presence of major cardiovascular risk factors, such as hypertension [19], diabetes mellitus [20], dyslipidemia [21], smoking, obesity and a sedentary lifestyle. Smoking was quantified as “pack years”, with a pack year being measured as 20 cigarettes being smoked daily for one year [22].

Medical data regarding demographics, personal medical history, tobacco, alcohol consumption habits and chronic medication were obtained from the observation charts. Body mass index (BMI) was calculated as the ratio of weight (kg) to height (m^2^).

A calibrated medical scale was used to assess the body weights of patients included in the study according to international standards. The measurement was performed for each patient on an unweighted basis, with patients removing clothing considered likely to generate significant weight fluctuations. The blood pressure profile was assessed in all patients enrolled in the study, with the main components used in the statistical analysis being systolic blood pressure (SBP, mmHg), diastolic blood pressure (DBP, mmHg) and pulse pressure (PP, mmHg).

The parameters of lipid (total cholesterol, low-density lipoprotein cholesterol, high-density lipoprotein cholesterol, and triglycerides) and carbohydrate profile (serum glucose), inflammatory markers (serum fibrinogen) and renal function (serum creatinine and urea) were evaluated. In addition to the biological parameters usually evaluated in all patients with associated cardiovascular pathologies, based on complete blood counts, we calculated a series of new biomarkers with proven roles, as shown in the literature, in the assessment of inflammatory status, such as NLR, PLR and LCR. NLR was calculated as the ratio of absolute neutrophil (N) to lymphocyte (L) values. PLR was calculated as the ratio of absolute platelets (P) to L values. LCR ratio was calculated as the ratio of absolute L to hs-CRP values. All results were presented according to the International System of Units.

#### 2.2.2. Transthoracic Echocardiography

Transthoracic echocardiography was performed at the first evaluation based on European guidelines (European Association of Cardiovascular Imaging) related to the purpose of the functional and morphological assessment of the heart [23]. All imaging examinations were performed using the same echocardiograph (Toshiba Aplio 500 Series, Toshiba Medical Systems Corporation, Ōtawara, Tochigi, Japan) by the same experienced cardiologist. Left ventricle (LV) systolic function was assessed by calculating the LV ejection fraction (LVEF) via the Simpson biplane method.

#### 2.2.3. Angiography

Peripheral angiography is the gold standard method of diagnosis and treatment in PAD. Prior to the procedure, all patients were informed of the risks and potential complications associated with the minimally invasive procedure. Biological samples were taken from all patients (especially renal function, complete blood count, blood group, and hemostasis parameters), and a venous line was fitted. In diabetic patients receiving Metformin treatment, it was recommended to discontinue treatment 24 h prior to the procedure and resume it after 48 h to reduce the risk of associated nephrotoxicity.

The angiography technique is the standard method used in all interventional cardiology centers [24,25]. Contrast medium injection was performed, allowing the visualization of the arterial system at the aorto-iliac, femuro-popliteal and infra-popliteal levels. In patients with stenotic lesions with indication of interventional revascularization, this procedure was performed in accordance with clinical protocols. All angiograms were performed by the same cardiologist. The severity of atherosclerotic lesions and their indication for interventional revascularization were determined using The Global Limb Anatomic Staging System (GLASS) [26]. The risk of amputation was assessed using the WIfl classification, taking into account the trophic lesions present, as well as ischemia or associated leg infections. The WIfl classification takes into account the presence of the three main components of trophic lesions, signs of ischemia and the presence of infection in the foot (scored from 0 to 3 points depending on their severity). As for the trophic lesion, it was quantified as follows: 0—rest pain, no ulcer; 1—small, superficial ulcer, located distal or at the level of the foot, without gangrene; 2—deep ulcer with exposure of bone, joint or tendon, possibly with gangrene limited to the toes; 3—deep, extensive ulcer affecting the calf, possibly with calcaneal or extensive gangrene. In case of ischemia, 0 points indicated an ABI greater than or equal to 0.8, an ankle BP greater than 100 mmHg and a halo BP greater than 60 mmHg; 1 indicated an ABI between 0.6 and 0.79, an ankle BP between 70 and 100 mmHg and a halo BP between 40 and 59 mmHg; 2 indicated an ABI between 0.4 and 0.59, an ankle BP between 50 and 70 mmHg and a halo BP between 30 and 39 mmHg; and 3 indicated an ABI below 0.4, an ankle BP below 50 mmHg and a halo BP below 30 mmHg. Foot infection was quantified as follows: 0—no signs or symptoms of infection; 1—local cutaneous and subcutaneous cellular tissue infection; 2—deeper local infection than the previous category; 3—systemic inflammation present [27].

### 2.3. Statistical Analysis

We used the Statistical Package for the Social Science (SPSS) statistics software (version 26 for Windows; SPSS Inc., Chicago, IL, USA) to perform statistical analysis of the parameters presented above. The results obtained were reported as mean ± standard deviation (SD) for the numerical parameters or frequency and percentages for categorical parameters. We tested the normal distribution of the data using the Kolmogorov–Smirnov test. The independent T-test and ANOVA (one way analysis of variance) were used to perform the analysis of continuous variables. Pearson’s and Spearman’s (r) correlation coefficients were used to test the reliability of statistically significant correlations identified in our study. A *p*-value of ≤0.05 was considered to be the threshold of statistical significance. The results are presented in Table 1 and Table 2. Receiver operating characteristic (ROC) analysis was performed to calculate the area under the curve for the biomarkers included in the study in order to identify predictors associated with severe obstructions. The Bonferroni Correction Method was used to perform multiple testing. Twelve tests were performed for each subgroup of patients, ensuring that for the data presented in Table 3, the *p*-value considered to be statistically significant was 0.0041.

### 2.4. Ethics

The study was approved by the Ethics Committee of the “Grigore T. Popa” University of Medicine and Pharmacy Iasi and the Ethics Committee of “St. Spiridon” Clinical Emergency Hospital, and it was conducted according to the Helsinki Declaration. All patients signed an informed consent statement, which mentioned that the results would be used for research purposes.

## 3. Results

In our study, we included 652 patients diagnosed with PAD (84.7% males, with a mean age of 66.46 ± 10.47 years old) who were evaluated in our clinic from an inflammatory point of view. According to the ankle–brachial index (ABI) value, we formed two study groups: patients with mild and moderate obstruction (judged as an ABI > 0.5) and patients with severe obstruction (with an ABI value below 0.5). We analyzed several demographic, hemodynamic, biochemical and imaging parameters, which are presented in Table 1.

In terms of demographic characteristics between the two groups, no significant differences in age (65.39 ± 11.06 vs. 67.18 ± 10.32, *p* = 0.333), gender (male patients 84.44% vs. 84.81%, *p* = 0.897) or residence (urban area 41.25% vs. 42.28%) were reported. In the case of anthropometric parameters, statistically significant differences were reported between the two groups analyzed, with patients with severe obstruction having higher mean weights (69.78 ± 9.66 vs. 81.20 ± 10.89, *p* = 0.048) and BMI scores (25.10 ± 2.99 vs. 27.15 ± 3.16 kg/m^2^, *p* = 0.053) than patients in the first group.

Of the vital parameters assessed, pulse pressure (71.54 ± 11.73 vs. 75.81 ± 14.93 mmHg, *p* < 0.001) was statistically significantly correlated with the degree of obstruction assessed using ABI. Patients with PAD enrolled in this study had a variety of comorbidities or cardiovascular risk factors. Cerebrovascular disease was more commonly present in patients with mild and moderate obstruction (8.95% vs. 7.09%, *p* = 0.387), but it was not a statistically significant parameter.

Smoking, which is one of the main risk factors associated with the development and progression of atherosclerotic lesions in patients with PAD, was more frequently associated with the group of patients with mild and moderate obstruction (71.60% vs. 63.54%, *p* = 0.033). Also, reporting the number of cigarettes smoked revealed a higher mean number of packs were smoked per year by patients with mild and moderate obstruction (25.63 ± 19.71 vs. 22.99 ± 18.94, *p* = 0.043) than those with an ABI less than 0.5.

Dyslipidemia was present in more than 50% of patients in both groups (*p* = 0.076), making it similar to dyslipidemia (hypercholesterolemia or hypertriglyceridemia, *p* > 0.05), hypertension (50.58% vs. 46.84%, *p* = 0.479) or class I obesity (67.7% vs. 73.2%, *p* = 0.762).

Regarding the number of risk factors, the majority of patients in both groups had associated two cardiovascular risk factors (32.7% vs. 39.1%, *p* = 0.041). Regarding lipid and carbohydrate profiles, no statistically significant differences were reported based on the severity of obstruction, as shown in Table 1.

Among the biological parameters, statistical analysis revealed statistically significant values for conventional and new inflammatory markers. The mean serum of high-sensitivity C-reactive protein (hs-CRP) (4.97 ± 3.01 mg/dL vs. 7.07 ± 3.83 mg/dL, *p* = 0.023) and serum fibrinogen levels (369.47 ± 115.96 vs. 414.71 ± 137.97, *p* = 0.001) were higher in patients with severe obstruction. The mean serum values of the inflammatory markers discussed in this paper were higher among patients with severe obstruction and considered to be statistically significant parameters in our group of patients (*p* = 0.037 for NLR, *p* = 0.041 for LCR and *p* = 0.019 for PLR).

The number and severity of atherosclerotic lesions in the vascular axis of lower limbs were assessed and quantified using angiography. Peripheral angiography predominantly identified a stenotic lesion in both groups (37.74% vs. 32.65%). The percentage of patients with more than 6 stenotic lesions identified was higher in patients with severe obstruction (4.27% vs. 5.83%, *p* < 0.001). Therapeutic management was performed in an integrative manner. In addition to drug treatment, a significant percentage of patients enrolled in the study received treatment via revascularization techniques. Interventional revascularization was preferred in patients with mild and moderate obstruction (12.06% vs. 4.30%, *p* < 0.001), while a higher percentage of patients with severe obstruction benefited from surgical revascularization (51.36% vs. 59.75%, *p* = 0.061). The risk of amputation was higher in patients with ABI values below 0.5 (24.12% vs. 37.47%, *p* < 0.001). Taking into account the staging based on the WIfl classification, patients with severe obstructive lesions had a higher mean score than PAD patients with mild and moderate atherosclerotic lesions (4.88 ± 0.54 vs. 5.37 ± 0.61, *p* = 0.047).

The main parameters of the lipid profile, carbohydrate profile and inflammatory markers were statistically analyzed based on the number of associated cardiovascular risk factors (Table 2). In patients with two associated risk factors, the serum fibrinogen level was found to be a statistically significant parameter, along with the evaluated inflammatory markers. In patients with more than three associated cardiovascular risk factors, patients with severe obstruction had higher mean serum values for NLR (3.75 ± 0.77 vs. 4.23 ± 1.01, *p* = 0.019), PLR (141,889 ± 74,258.71 vs. 149,663.04 ± 76,752.19, *p* = 0.042) and LCR (7.86 ± 11.17 vs. 8.50 ± 11.89, *p* = 0.007), which were associated with a more pronounced inflammatory state.

Patients with gangrene are frequently associated with a high titer of inflammatory markers, which is why we decided to perform an analysis of the subgroup of patients without gangrene and with serum hs-CRP values below 10 mg/dL (first group—29 patients with gangrene and 21 patients with hs-CRP values above the mentioned limit; second group—92 patients with gangrene and 39 patients with hs-CRP values above 10 mg/dL; final analysis: 207 patients vs. 264 patients with PAD).

**Figure 2:**
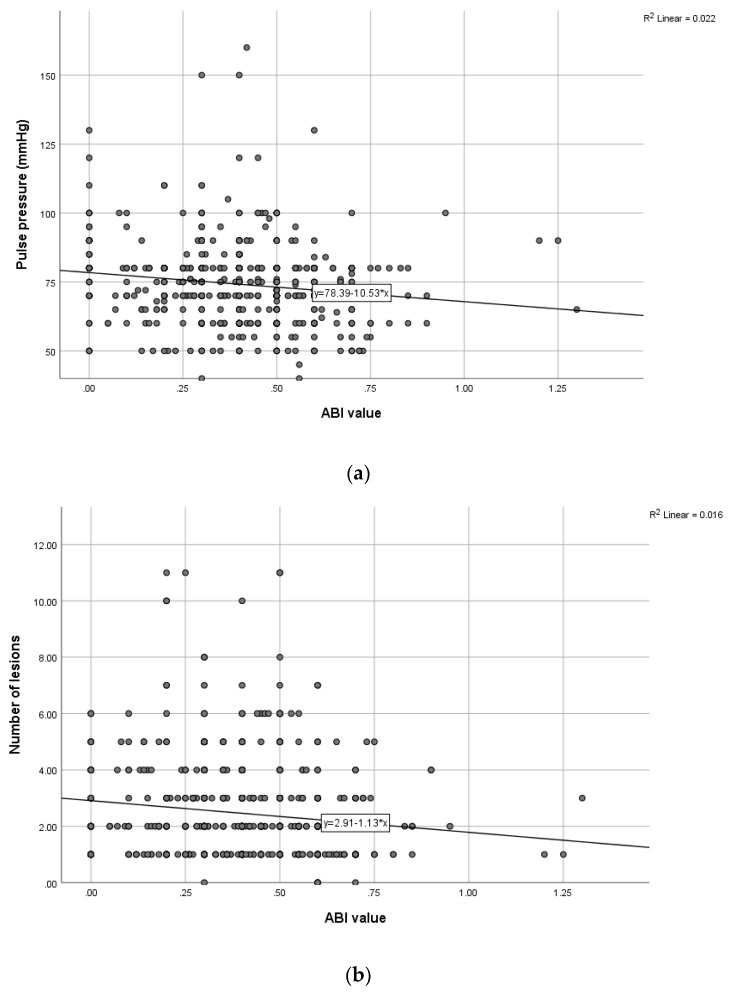
Correlation between ABI and pulse pressure (**a**) or the number of lesions (**b**) (ABI: ankle–brachial index).

**Figure 3:**
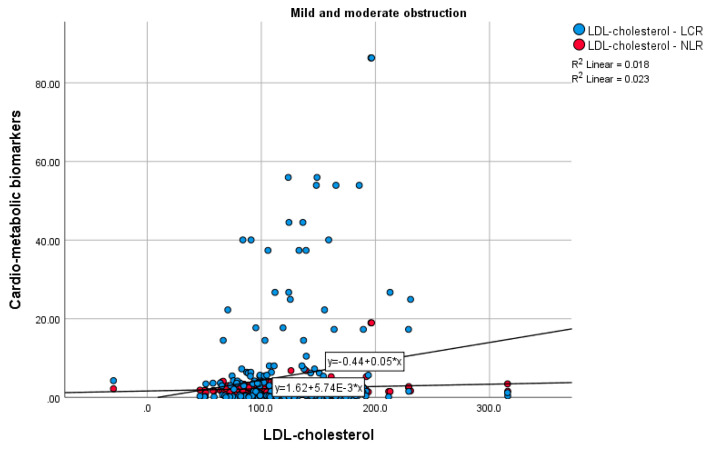
Correlation between LDL cholesterol, NLR and LCR in patients with mild and moderate obstruction. (LDL: low-density lipoproteins NLR: neutrophil-to-lymphocyte ratio; LCR: lymphocyte-to-C-reactive protein ratio).

We identified several statistically significant correlations (after adjusting for various co-founders, such as age, anthropometric parameters or the presence of gangrene) in our study group, as shown in Table 3. Among the lipid profile parameters, LDL cholesterol had a direct proportional association with all three proposed biomarkers in patients with severe obstruction. NLR was statistically significantly correlated with the number of cardiovascular risk factors present in both patients with mild or moderate obstruction (*p* = 0.0006) and those with severe obstruction (*p* = 0.0004). The number of angiographically detected atherosclerotic lesions was also statistically significantly correlated with NLR in patients included in the first group (*p* = 0.0007), as well as with NLR (*p* = 0.0005). LCR (*p* = 0.0014) and PLR (*p* = 0.0004) were statistically significantly correlated with patients in the second group. The risk of amputation was assessed in all patients enrolled in this study, with statistically significant correlations noted between its presence and NLR (*p* = 0.0005), LCR (*p* = 0.0012) and PLR (*p* = 0.0010) in the group of patients with ABI values below 0.5 (Figure 2 and Figure 3). The predictive value of NLR and PLR was also demonstrated using univariate and multivariate statistical analysis, as shown in Table 4.

**Figure 4:**
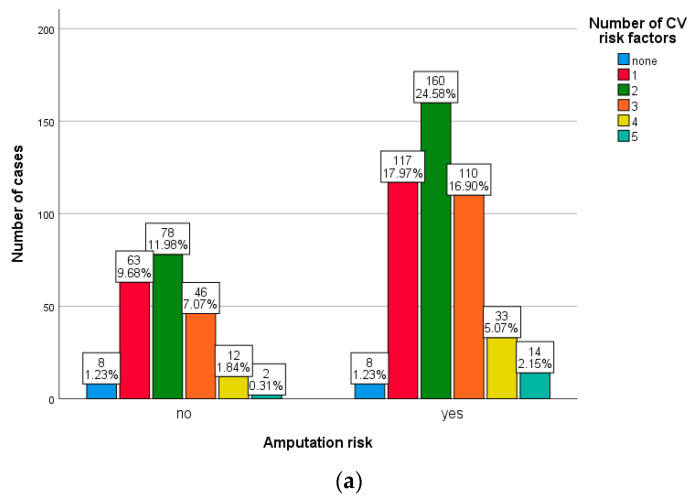

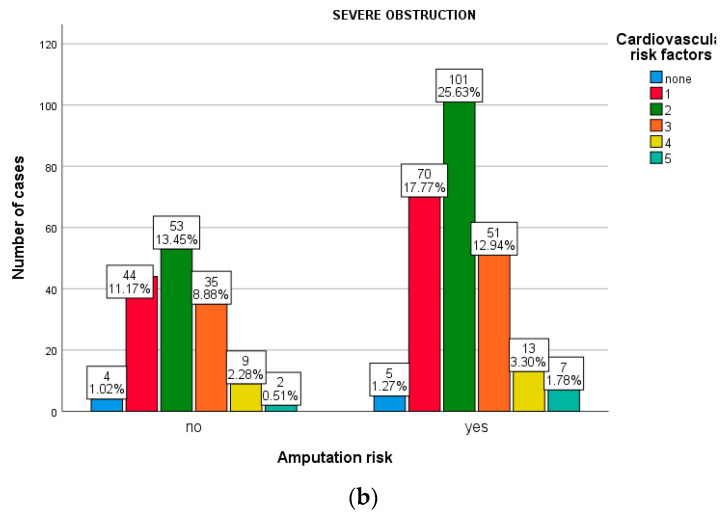
Weight of cardiovascular risk factors according to the risk of amputation in patients with mild and moderate (**a**) or severe obstruction (**b**).

We evaluated the weights of cardiovascular risk factors in the two groups of patients and observed that the majority of patients presented two risk factors (24.58% vs. 26.63%, *p* = 0.194 for the first group and *p* = 0.804 for the second group). Patients with mild and moderate obstruction, as well as those with ABI values below 0.5, showed statistically significant correlations between the risk of amputation and the presence of gangrene or intermittent claudication at rest (*p* < 0.001 for all associations) (Figure 4).

**Figure 5:**
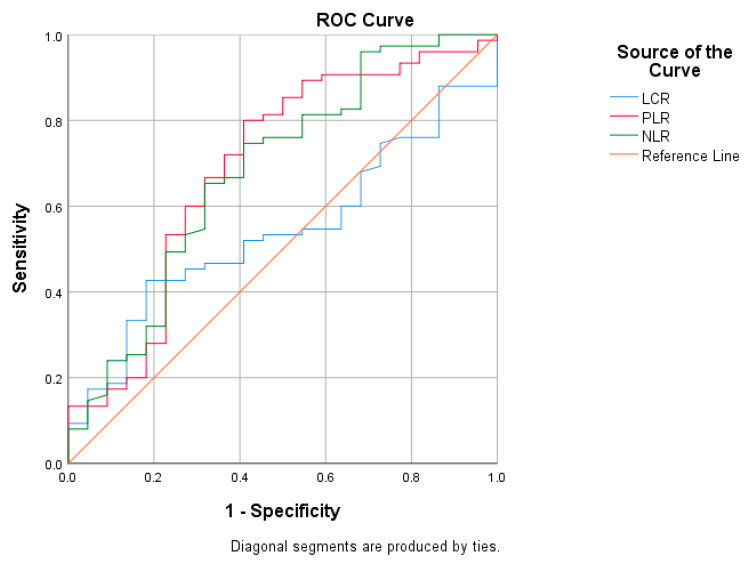
The area under the curve of the receiver operating characteristic used to determine inflammatory biomarkers in patients with amputation risk and severe obstruction (AUC: area under the curve, NLR: neutrophil-to-lymphocyte ratio; LCR: lymphocyte-to-C-reactive protein ratio, PLR: platelet-to-lymphocyte ratio).

**Figure 6:**
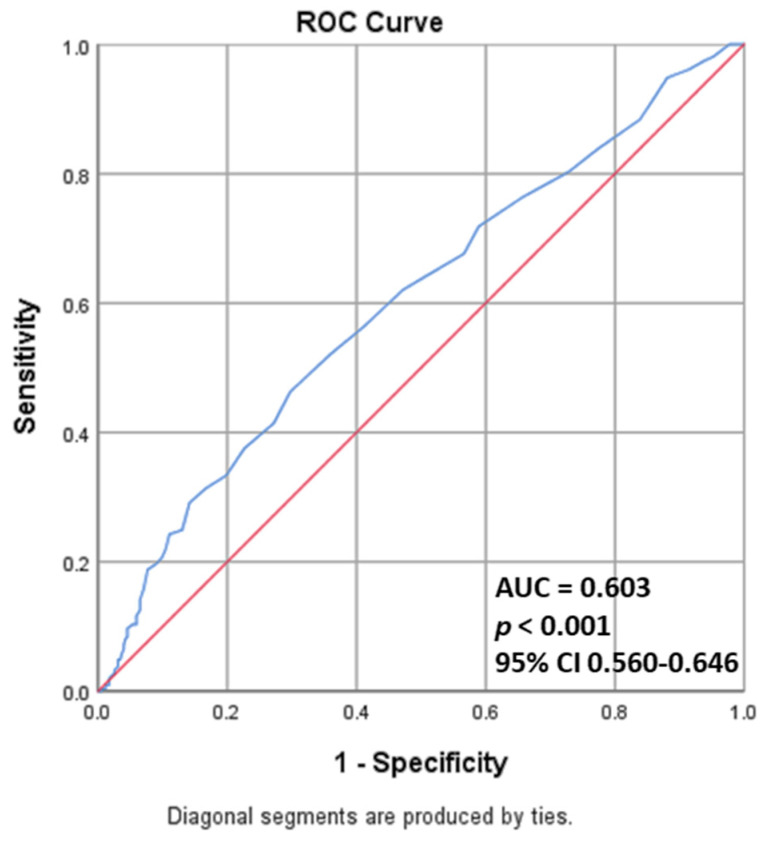
The area under the curve of the receiver operating characteristic hs-CRP in patients with amputation risk and severe obstruction (AUC: area under the curve; CI: confidence interval).

The respective values of NLR, LCR and PLR predictors associated with amputation risk in patients with severe obstruction were via receiver operating characteristic (ROC) analysis (Figure 5). NLR (area under the curve <AUC> = 0.682, *p* = 0.010, 95% confidence interval <CI> 0.419–0.664) and PLR (AUC = 0.692, *p* = 0.006, 95% CI 0.556–0.829) are inflammatory markers associated with a high risk of amputation, while LCR (AUC = 0.541, *p* = 0.558, 95% CI 0.419–0.664) did not prove its value as a predictor in our study. In addition to the markers presented above, we tested the predictive value associated with amputation risk for hs-CRP, with this classic marker being a statistically significant predictor in our study group, as shown in Figure 6.

## 4. Discussion

Atherosclerosis is a multifactorial process in which inflammatory status plays a role in determining both the appearance of lesions and their progression and, therefore, in increasing the risk of an acute cardiovascular event [28,29]. In total, 84.7% of the patients enrolled in our study were males, with a mean age of 66.46 ± 10.47 years. By analyzing a broad spectrum of parameters, we highlighted the role played by the proposed inflammatory biomarkers (NLR, PLR, LCR) in the management of PAD and their prognostic implications.

Various degrees of inflammation have been identified at all stages of PAD, with this association also being thoroughly researched. Some studies indicate a stronger association with PAD than with coronary artery disease, suggesting the presence of different predominant substrates [30]. In our study, serum hs-CRP levels were elevated, regardless of the severity of obstruction, representing a statistically significant inflammatory marker (*p* = 0.023). Also, regarding medium- and long-term evolution of hs-CRP, it has been shown that in patients with PAD, high levels at the time of the first revascularization intervention and their persistence at 3.6 years of follow-up are associated with an independent increase in all-cause, cardiovascular and malignancy-related mortality, with these results being supported by other similar research [31,32].

The mean serum values of NLR (*p* = 0.037), LCR (*p* = 0.041) and PLR (*p* = 0.019) were higher in patients with severe obstruction, as well as statistically significant biomarkers in our analyzed group. Similar results have been reported by other investigators in the literature, with the calculation of these biological parameters having both therapeutic and prognostic value.

The role played by NLR as a predictive factor in assessing the risk of death or an acute cardiovascular event has been extensively reviewed in the literature [33,34,35], and the reported results are similar to those obtained in this study. Positive and statistically significant correlations were found between NLR, LDL cholesterol, fasting glucose and the number of cardiovascular risk factors, as well as the presence of gangrene and the number of atherosclerotic lesions angiographically identified. A meta-analysis including 38 clinical studies summarizing 76,000 patients demonstrated that high NLR values increased the risk of coronary artery disease (CAD) 1.62-fold and the risk of stroke 3.86-fold, which justifies regular evaluation of the complete blood count [36].

Patients with PAD are associated with a high risk of developing an acute vascular event in the absence of integrative management [37]. A group of Romanian researchers demonstrated that high NLR and PLR values are associated with an 11-fold increase in the risk of amputation and a 22-fold increase in the risk of death in patients with acute limb ischemia [8]. Similar results were reported by Coelho et al. [38], who analyzed a group of 345 patients with acute inferior ischemia and demonstrated that an NLR value above 5.4 is consistent with a sensitivity of 90.5% and specificity of 73.6% for the occurrence of death at 30 days or amputation. PAD patients with obstructive atherosclerotic lesions at the femuro-popliteal level who undergo peripheral revascularization surgery and have associated high pre-operative NLR and PLR values have an increased risk of primary patency failure at 12 months after revascularization [39]. Erturk et al. [40] analyzed a group of 593 patients with occlusive PAD and divided them into two groups according to the NLR value (below 3 and above 3), observing that age and NLR values above three are independent factors associated with long-term mortality in these patients.

Cosarca et al. [7] demonstrated that NLR values above 3.48 have a sensitivity of 60% and a specificity of 72.44% regarding the need for amputation after revascularization in patients with PAD, thus making them a useful pre-operative prognostic marker. Similarly, the same group of investigators demonstrated in PLR that serum values above 152 are associated with a sensitivity of 54.17% and a specificity of 71.79% regarding amputation. Increased absolute neutrophil counts relative to lymphocyte counts are associated with a poorer prognosis in PAD patients undergoing interventional revascularization [41,42].

Similar to the NLR, the PLR has a predictive value regarding the risk of an acute vascular event; in the case of patients with PAD, the existence of a value of more than 150 is associated with a relative risk about two times higher than that of critical atherosclerotic lesions [43]. Liu et al. [44] analyzed a cohort of 355 diabetic patients, in whom they assessed the risk of developing PAD and identified NLR and PLR as predictors associated with the development and progression of atherosclerotic processes in this category of patients, finding evidence of the superiority of PLR.

The validity of PLR’s use as an inflammatory marker is secondary to the pro-inflammatory effect exerted by platelets [45]. Initially investigated in various oncological clinical trials [46], this biomarker has increasingly broad validity as a predictor of moderate-to-severe functional decline in PAD patients, as demonstrated above. PLR is another biomarker that plays a prognostic role in the management of patients with PAD, with elevated titers being associated with a high risk of critical ischemia or acute vascular events (odds ratio of 1.9 for PLR > 150) [43].

PLR also modulates the risk of death among patients with PAD. Uzun et al. [47] demonstrated through the analysis of a cohort of 602 patients with PAD that the identification of a PLR value above 142 is an independent predictor of an increased long-term risk of death.

In addition to the biomarkers mentioned above, the monocyte-to-HDL cholesterol ratio was analyzed in relation to the severity and prognosis of PAD, but the reported results have so far been contradictory [48]. Clinical studies reported in the literature that do not report superior results for this inflammatory marker compared to NLR also exist [48]. On the other hand, Selvaggio et al. [10] reported the existence of a directly proportional relationship between increases in PLR and the monocyte-to-HDL cholesterol ratio and decrease in ABI (*p* = 0.0011). Guetl et al. [49] conducted a retrospective study in which 2121 patients with PAD were included and, using multivariate regression statistical analysis, demonstrated that increased WMR values (odds ratio 2.25, *p* < 0.001), older age (odds ratio 1.05, *p* < 0.001), elevated CRP titer (odds ratio 1.01, *p* < 0.001) and diabetes mellitus (odds ratio 2.38, *p* < 0.001) were independently significant predictors of chronic limb-threatening ischemia occurrence.

Gary et al. [50] demonstrated that patients with NLR values above 3.95 have a 2.5-fold increased risk of critical lower limb injury, making this inflammatory biomarker an easily measured prognostic parameter that can be used in everyday practice. Neutrophilia is responsible for increasing the value of the ratio, being the result of various pathophysiological processes that contribute to the maintenance of the pro-inflammatory status in PAD [51]. Taşoğlu et al. [52] showed that the presence of an NLR value above 3.2 and a PLR above 160 are associated with a high risk of amputation, with the average duration being about 2 years to date.

A significant percentage of patients with associated PAD and CAD had this condition as an issue secondary to existing atherosclerotic damage, which was sometimes subclinical in nature. In this category of patients, Arbel et al. [53] demonstrated that an NLR value above three is associated with a relative risk of 2.45 regarding the existence of sub-occlusive coronary lesions, as well as the occurrence of an acute cardiovascular event in the next 3 years (odds ratio: 1.55). Yuan et al. [54] analyzed a cohort of 235 patients with COPD and demonstrated a positive correlation between NLR and WBC, hs-CRP, BMI and 6-min walking test distance, thus making it an indicator of muscle function in this category of patients. Interruption of regular physical training also produced a number of negative changes in inflammatory parameters, with a 48.2% increase in NLR reported in the clinical study by Liao et al. [55].

Our study presents several limitations due to the lack of follow-up. The heterogeneity of the study group or the potential risk associated with the inclusion of patients with elevated serum CRP values due to associated infections are additional aspects that may influence the obtained results. We excluded records in which medical data were unavailable. This step was taken to minimize the risk of misclassification, introducing a limited risk of selection bias.

Our future research direction will be to investigate the influence of the proposed markers (NLR, PLR, CSF) on the predictive value of amputation risk in relation to a series of biochemical or clinical models, such as PREVENT III or the BASIL model, that exist in the literature [56].

## 5. Conclusions

In our study, we demonstrated the predictive value of the analyzed inflammatory biomarkers and the importance of their assessment in patients with severe obstruction and a high risk of amputation. NLR and PLR are predictors used in patients with ABI values below 0.5 and a risk of amputation, thus making them parameters with both therapeutic and prognostic value. NLR, PLR and WMR are easy-to-determine and reproducible parameters, which can be easily used in daily practice, as they also have therapeutic and prognostic value among patients with PAD.

## Figures and Tables

**Table 1:** Demographics, anthropometric parameters, biological data and exercise stress test parameters.

Parameter	Total Group (*n* = 652)	Mild and Moderate Obstruction (*n* = 257)	Severe Obstruction (*n* = 395)	*p*
**Demographics**				
Age	66.46 ± 10.47	65.39 ± 11.06	67.18 ± 10.32	0.333
Males	552 (84.7%)	217 (84.44%)	335 (84.81%)	0.897
Area of residence—urban	273 (41.9%)	106 (41.25%)	167 (42.28%)	0.794
**Anthropometric data**				
Height. M	1.92 ± 6.4	1.67 ± 0.05	2.06 ± 7.98	0.435
Weight. Kg	75.94 ± 9.15	69.78 ± 9.66	81.20 ± 10.89	0.048
BMI. Kg/m^2^	26.21 ± 3.01	25.10 ± 2.99	27.15 ± 3.16	0.053
**Vitals**				
HR. bpm	74.12 ± 13.96	73.87 ± 14.55	75.11 ± 16.38	0.076
Systolic BP. mmHg	141.93 ± 14.89	140.78 ± 15.05	142.49 ± 14.37	0.069
Diastolic BP. mmHg	80.15 ± 7.66	79.54 ± 8.34	80.58 ± 7.03	0.068
Mean BP. mmHg	100.74 ± 8.93	99.96 ± 9.36	101.22 ± 8.40	0.040
Pulse pressure. mmHg	73.56 ± 12.99	71.54 ± 11.73	75.81 ± 14.93	<0.001
**Cardiovascular risk factors & comorbidities**				
Smoking	435 (66.72%)	184 (71.60%)	251 (63.54%)	0.033
Smoking—packs smoked per year	23.69 ± 18.43	25.63 ± 19.71	22.99 ± 18.94	0.043
Dyslipidemia	350 (53.68%)	149 (57.98%)	201 (50.89%)	0.076
Diabetes mellitus	213 (32.67%)	93 (36.19%)	120 (30.38%)	0.226
Hypercholesterolemia (>200 mg/dL)	267 (40.95%)	113 (43.97%)	154 (38.99%)	0.206
Hypercholesterolemia (>250 mg/dL)	67 (10.28%)	28 (10.89%)	39 (9.87%)	0.675
HDL cholesterol < 40 mg/dL	244 (37.42%)	98 (38.13%)	80 (20.25%)	0.949
LDL cholesterol > 130 mg/dL	276 (42.33%)	111 (43.19%)	165 (41.77%)	0.720
Hypertriglyceridemia	35 (5.37%)	15 (5.84%)	20 (5.06%)	0.857
Overweight	51 (70.8%)	21 (67.7%)	30 (73.2%)	0.762
Obesity class I	18 (25.0%)	9 (29.0%)	9 (22.0%)	
Obesity class II	3 (4.2%)	1 (3.2%)	2 (4.9%)	
Hypertension	315 (48.31%)	130 (50.58%)	185 (46.84%)	0.479
Number of risk factors				
0	16 (2.5%)	7 (2.7%)	9 (2.3%)	0.041
1	180 (27.6%)	66 (25.7%)	114 (28.9%)	
2	238 (36.6%)	84 (32.7%)	154 (39.1%)	
3	156 (24.0%)	70 (27.2%)	86 (21.8%)	
4	45 (6.9%)	23 (8.9%)	22 (5.6%)	
5	16 (2.5%)	7 (2.7%)	9 (2.3%)	
Cerebrovascular disease	51 (7.82%)	23 (8.95%)	28 (7.09%)	0.387
**Biological data**				
Total cholesterol. mg/dL	198.47 ± 46.57	196.92 ± 46.65	194.36 ± 45.85	0.606
LDL cholesterol. mg/dL	126.82 ± 40.30	127.19 ± 40.70	126.50 ± 39.91	0.607
HDL cholesterol. mg/dL	41.65 ± 10.60	41.79 ± 11.33	41.25 ± 9.94	0.949
Triglycerides. mg/dL	135.45 ± 71.79	139.67 ± 80.04	133.06 ± 65.98	0.791
Serum creatinine. mg/dL	0.96 ± 0.36	1.07 ± 0.38	1.05 ± 0.36	0.973
Serum urea. mg/dL	44.71 ± 18.92	46.31 ± 21.70	43.74 ± 17.60	0.297
Creatinine clearance. mL/min/1.73 m^2^	62.49 ± 22.01	62.84 ± 22.68	62.16 ± 21.47	0.685
Fasting glucose. mg/dL	118.49 ± 48.90	121.93 ± 49.97	117.32 ± 48.75	0.186
Serum fibrinogen. mg/dL	395.59 ± 132.22	369.47 ± 115.96	414.71 ± 137.97	0.001
hs-CRP. mg/dL	6.34 ± 2.78	4.97 ± 3.01	7.07 ± 3.83	0.023
Hematocrit. %	41.74 ± 5.16	41.43 ± 5.47	41.85 ± 5.17	0.125
Platelets (×10^3^/mL)	297.44 ± 11.17	281.73 ± 94.37	308.63 ± 119.14	0.018
NLR	3.21 ± 2.16	2.17 ± 1.75	4.20 ± 0.89	0.037
LCR	8.75 ± 13.22	6.21 ± 12.34	7.15 ± 16.37	0.041
PLR	138,280 ± 71,678.82	133,560.11 ± 60,047.82	145,181.91 ± 70,236.67	0.019
**Clinical parameters**				
Pain at rest	541 (81.44%)	191 (74.32%)	350 (88.61%)	<0.001
Erythema	77 (11.81%)	30 (11.67%)	47 (11.90%)	0.930
Ulcerations	93 (14.26%)	34 (13.23%)	59 (14.94%)	0.542
Necrosis	27 (4.14%)	6 (2.33%)	21 (5.32%)	0.062
Gangrene	121 (18.51%)	29 (11.28%)	92 (23.29%)	0.001
Bilateral clinical involvement	231 (35.43%)	82 (31.91%)	149 (37.72%)	0.052
Cardiac murmurs	119 (18.25%)	51 (19.84%)	68 (17.22%)	0.271
Femoral artery murmur	149 (22.85%)	50 (19.46%)	99 (25.06%)	0.175
Carotid artery murmur	77 (11.81%)	34 (13.23%)	43 (10.89%)	0.484
Renal artery murmur	24 (3.68%)	9 (3.50%)	15 (3.80%)	0.707
**Rutherford classification**				
Class 3	106 (16.3%)	66 (25.7%)	40 (10.2%)	<0.001 *
Class 4	213 (32.7%)	89 (34.6%)	123 (31.2%)	
Class 5	205 (31.4%)	65 (25.3%)	140 (35.5%)	
Class 6	128 (19.6%)	37 (14.4%)	91 (23.1%)	
**Leriche–Fontaine classification**				
2nd stage	105 (16.1%)	64 (24.9%)	41 (10.13%)	<0.001 *
3rd stage	212 (32.5%)	121 (47.08%)	91 (23.03%)	
4th stage	335 (51.4%)	72 (28.01%)	263 (66.58%)	
**Paraclinical data**				
Arterial Doppler US	110 (16.95%)	28 (10.89%)	81 (20.51%)	0.004
Angio MRI	32 (4.9%)	10 (3.89%)	22 (5.57%)	0.448
Arteriography	635 (97.4%)	252 (98.05%)	382 (96.71%)	0.305
Number of lesions (stenosis and thrombosis)				<0.001
0	5 (0.8%)	4 (1.56%)	4 (0.25%)	
1	226 (34.7%)	97 (37.74%)	129 (32.65%)	
2	183 (28.1%)	78 (30.35%)	105 (26.58%)	
3	98 (15.0%)	40 (15.56%)	58 (14.68%)	
4	65 (10.0%)	15 (5.84%)	50 (12.66%)	
5	40 (6.1%)	11 (4.28%)	29 (7.34%)	
≥6	35 (4.6%)	10 (4.27%)	23 (5.83%)	
LVEF. %	57.36 ± 10.08	58.20 ± 9.95	56.66 ± 10.17	0.057
**Therapeutic management**				
Medical	650 (99.8%)	257 (100.0%)	393 (99.49%)	0.521
Interventional revascularization	48 (7.36%)	31 (12.06%)	17 (4.30%)	<0.001
Surgical revascularization	369 (56.6%)	132 (51.36%)	236 (59.75%)	0.061
Risk of amputation	210 (32.1%)	62 (24.12%)	148 (37.47%)	<0.001

All values are expressed as mean ± standard deviation (SD). LDL cholesterol: low-density lipoprotein cholesterol, HDL cholesterol: high-density lipoprotein cholesterol; hs-CRP: high-sensitivity C-reactive protein (normal range 0–1 mg/dL); HbA1C: glycated hemoglobin; NLR: neutrophil-to-lymphocyte ratio, normal range 0.43–2.75 in males and 0.37–2.87 in females; LCR: lymphocyte-to-C-reactive protein ratio, normal range—not defined; PLR: platelet-to-lymphocyte ratio, normal range 36.63–149.13 in males and 43.36–172.68 in females; HR: heart rate; BP: blood pressure; ABI: ankle–brachial index; US: ultrasonography; MRI: magnetic resonance imaging; LVEF: left ventricle ejection fraction. * The *p*-value was assessed on the basis of walking distances until the onset of intermittent claudication.

**Table 2:** Biological parameters based on the number of associated cardiovascular risk factors.

Parameter	Mild and Moderate Obstruction (*n* = 257)	Severe Obstruction (*n* = 395)	*p*
**No Cardiovascular Risk Factors**			
Total cholesterol, mg/dL	202.11 ± 28.03	199.86 ± 46.12	0.882
LDL cholesterol, mg/dL	136.31 ± 22.56	125.89 ± 35.95	0.806
HDL cholesterol, mg/dL	41.78 ± 3.99	40.29 ± 11.28	0.612
Triglycerides, mg/dL	120.11 ± 42.08	168.43 ± 66.50	0.144
Serum creatinine, mg/dL	0.92 ± 0.15	1.20 ± 0.71	0.420
Serum urea, mg/dL	38.56 ± 13.68	55.86 ± 45.27	0.057
Fasting glucose, mg/dL	90.44 ± 7.54	95.43 ± 8.72	0.436
Serum fibrinogen, mg/dL	406.11 ± 107.53	478.57 ± 136.37	0.624
NLR	2.22 ± 1.89	2.46 ± 2.04	0.169
LCR	6.05 ± 11.63	7.17 ± 11.78	0.247
PLR	134,115 ± 59,753.70	138,098 ± 71,367.71	0.079
**1 cardiovascular risk factor**			
Total cholesterol, mg/dL	203.61 ± 55.30	198.61 ± 46.03	0.576
LDL cholesterol, mg/dL	133.90 ± 48.42	129.50 ± 40.98	0.690
HDL cholesterol, mg/dL	42.03 ± 10.85	41.70 ± 10.11	0.497
Triglycerides, mg/dL	138.39 ± 62.06	137.03 ± 65.79	0.772
Serum creatinine, mg/dL	1.01 ± 0.42	1.06 ± 0.42	0.399
Serum urea, mg/dL	41.95 ± 17.85	43.20 ± 20.82	0.785
Fasting glucose, mg/dL	96.25 ± 24.22	98.65 ± 20.82	0.113
Serum fibrinogen, mg/dL	396.48 ± 145.22	361.08 ± 118.96	0.135
NLR	2.31 ± 1.67	2.43 ± 2.89	0.083
LCR	5.98 ± 10.85	6.48 ± 9.45	0.062
PLR	129,774.51 ± 53,782.3	134,781 ± 51,741	0.108
**2 cardiovascular risk factors**			
Total cholesterol, mg/dL	191.23 ± 44.57	203.15 ± 49.21	0.457
LDL cholesterol, mg/dL	124.63 ± 36.81	130.56 ± 43.13	0.848
HDL cholesterol, mg/dL	40.74 ± 9.02	41.86 ± 14.25	0.749
Triglycerides, mg/dL	129.31 ± 66.12	153.67 ± 111.67	0.168
Serum creatinine, mg/dL	1.05 ± 0.34	1.05 ± 0.35	0.754
Serum urea, mg/dL	43.94 ± 18.28	45.12 ± 18.11	0.801
Fasting glucose, mg/dL	115.73 ± 50.02	112.27 ± 41.37	0.064
Serum fibrinogen, mg/dL	430.95 ± 137.66	349.19± 99.69	<0.001
NLR	3.01 ± 2.48	3.99 ± 2.91	0.048
LCR	7.69 ± 10.02	8.23 ± 9.34	0.031
PLR	135,667.73 ± 51,589	14,478.67	0.005
**≥3 cardiovascular risk factors**			
Total cholesterol, mg/dL	189.03 ± 36.78	190.36 ± 44.70	0.326
LDL cholesterol, mg/dL	121.03 ± 34.73	122.93 ± 38.87	0.157
HDL cholesterol, mg/dL	41.18 ± 10.57	41.90 ± 9.30	0.778
Triglycerides, mg/dL	134.09 ± 71.26	127.65 ± 51.23	0.239
Serum creatinine, mg/dL	1.09 ± 0.31	1.08 ± 0.36	0.537
Serum urea, mg/dL	45.64 ± 16.60	48.69 ± 22.67	0.098
Fasting glucose, mg/dL	142 ± 55.38	147.26 ± 55.54	0.099
Serum fibrinogen, mg/dL	411.74 ± 132.16	384.41 ± 120.66	0.173
NLR	3.75 ± 0.77	4.23 ± 1.01	0.019
LCR	7.86 ± 11.17	8.50 ± 11.89	0.007
PLR	141,889.12 ± 74,258.71	149,663.04 ± 76,752.19	0.042

**Table 3:** Correlations between inflammatory markers and demographic, anthropometric or clinical–paraclinical parameters.

Mild and Moderate Obstruction (*n* = 207)							Severe Obstruction (*n* = 264)					
	NLR		LCR		PLR		NLR		LCR		PLR	
	r	*p*	r	*p*	r	*p*	r	*p*	r	*p*	r	*p*
Total cholesterol	0.085	0.0049	0.098	0.0033	0.025	0.0190	0.002	0.0268	0.022	0.0184	0.009	0.0240
LDL cholesterol	0.134	0.0009	0.151	0.0004	0.063	0.0088	0.572	0.0005	0.626	0.0012	0.715	0.0010
HDL cholesterol	−0.027	0.0185	−0.03	0.0177	−0.076	0.0063	−0.038	0.0125	−0.055	0.0078	−0.033	0.0141
Triglycerides	−0.076	0.0063	−0.078	0.0059	−0.031	0.0171	0.023	0.0182	0.039	0.0122	0.011	0.0232
Fasting glucose	0.007	0.0252	−0.043	0.0137	−0.11	0.0240	0.416	0.0005	0.062	0.0061	−0.014	0.0217
Pulse pressure	−0.007	0.0253	−0.017	0.0217	−0.038	0.0153	0.029	0.0157	0.026	0.0168	0.008	0.0243
Smoking—packs smoked per year	0.113	0.0019	0.047	0.0125	−0.009	0.0246	0.012	0.0226	−0.014	0.0219	0.04	0.0259
Number of risk factors	0.317	0.0006	0.598	0.0017	0.921	0.0003	0.219	0.0004	0.468	0.0012	0.711	0.0007
Pain at rest	0.817	0.0013	0.643	0.0028	0.753	0.0009	0.416	0.0013	0.37	0.0014	0.446	0.0005
Number of lesions	−0.77	0.0007	−0.296	0.0018	−0.538	0.0016	0.796	0.0005	0.234	0.0014	0.505	0.0004
LVEF (%)	0.065	0.0083	0.039	0.0148	0.426	0.0071	−0.024	0.0178	−0.015	0.0213	−0.083	0.0028
Risk of amputation	0.158	0.0024	0.227	0.0023	0.301	0.0024	0.712	0.0005	0.331	0.0012	0.488	0.0010

r: Pearson’s correlation; LDL: low-density lipoproteins; HDL: high-density lipoprotein; BMI: body mass index; NLR: neutrophil-to-lymphocyte ratio; LCR: lymphocyte-to-CRP ratio; PLR: platelet-to-lymphocyte ratio; LVEF: left ventricle ejection fraction.

**Table 4:** Univariate and multivariate statistical analysis for NLR, PLR and LCR among patients with severe obstruction.

Parameter	Univariate Regression			Multivariate Regression		
	*β*	*p*	Odds Ratio (95% CI)	*β*	*p*	Odds Ratio (95% CI)
LCR	0.043	0.015	1.051 (1.009–1.085)			
NLR	0.179	<0.001	1.292 (1.131–1.290)	0.025	0.029	1.054 (1.005–1.105)
PLR	0.033	0.002	1.053 (1.011–1.044)	0.525	0.005	0.591 (0.410–0.852)

## Data Availability

All the data are available from the corresponding author upon reasonable request.

## References

[1] Shamaki G.R., Markson F., Soji-Ayoade D., Agwuegbo C.C., Bamgbose M.O., Tamunoinemi B.-M. (2022). Peripheral Artery Disease: A Comprehensive Updated Review. Curr. Probl. Cardiol..

[2] Aday A.W., Matsushita K. (2021). Epidemiology of Peripheral Artery Disease and Polyvascular Disease. Circ. Res..

[3] Campia U., Gerhard-Herman M., Piazza G., Goldhaber S.Z. (2019). Peripheral Artery Disease: Past, Present, and Future. Am. J. Med..

[4] Colantonio L.D., Hubbard D., Monda K.L., Mues K.E., Huang L., Dai Y., Jackson E.A., Brown T.M., Rosenson R.S., Woodward M. (2020). Atherosclerotic Risk and Statin Use Among Patients with Peripheral Artery Disease. J. Am. Coll. Cardiol..

[5] Libby P. (2021). The Changing Landscape of Atherosclerosis. Nature.

[6] Björkegren J.L.M., Lusis A.J. (2022). Atherosclerosis: Recent Developments. Cell.

[7] Cosarca M.C., Hălmaciu I., Muresan A.V., Suciu B.A., Molnar C., Russu E., Horvath E., Niculescu R., Puscasiu L., Bacalbaşa N. (2022). Neutrophil-to-lymphocyte, Platelet-to-lymphocyte and Lymphocyte-to-monocyte Ratios Are Associated with Amputation Rates in Patients with Peripheral Arterial Disease and Diabetes Mellitus Who Underwent Revascularization: A Romanian Regional Center Study. Exp. Ther. Med..

[8] Arbănași E.M., Mureșan A.V., Coșarcă C.M., Kaller R., Bud T.I., Hosu I., Voidăzan S.T., Arbănași E.M., Russu E. (2022). Neutrophil-to-Lymphocyte Ratio and Platelet-to-Lymphocyte Ratio Impact on Predicting Outcomes in Patients with Acute Limb Ischemia. Life.

[9] Yang Y.-L., Wu C.-H., Hsu P.-F., Chen S.-C., Huang S.-S., Chan W.L., Lin S.-J., Chou C.-Y., Chen J.-W., Pan J.-P. (2020). Systemic Immune-Inflammation Index (SII) Predicted Clinical Outcome in Patients with Coronary Artery Disease. Eur. J. Clin. Investig..

[10] Selvaggio S., Abate A., Brugaletta G., Musso C., Di Guardo M., Di Guardo C., Vicari E.S.D., Romano M., Luca S., Signorelli S.S. (2020). Platelet-to-lymphocyte Ratio, Neutrophil-to-lymphocyte Ratio and Monocyte-to-HDL Cholesterol Ratio as Markers of Peripheral Artery Disease in Elderly Patients. Int. J. Mol. Med..

[11] Okan S. (2020). The Relationship between Exercise Capacity and Neutrophil//Lymphocyte Ratio in Patients Taken to Cardiopulmonary Rehabilitation Program. Bratisl. Lek. Listy.

[12] Kaya B.B., Özbilgin N. (2019). Effect of Cardiac Rehabilitation on Mortality Related Inflammatory Markers. J. Surg. Med..

[13] Grigorescu E.D., Sorodoc V., Floria M., Anisie E., Popa A.D., Onofriescu A., Ceasovschih A., Sorodoc L. (2019). The Inflammatory Marker hsCRP as a Predictor of Increased Insulin Resistance in Type 2 Diabetics without Atherosclerotic Manifestations. Rev. Chim..

[14] Nidorf S.M., Fiolet A.T.L., Mosterd A., Eikelboom J.W., Schut A., Opstal T.S.J., The S.H.K., Xu X.-F., Ireland M.A., Lenderink T. (2020). Colchicine in Patients with Chronic Coronary Disease. N. Engl. J. Med..

[15] Tardif J.-C., Kouz S., Waters D.D., Bertrand O.F., Diaz R., Maggioni A.P., Pinto F.J., Ibrahim R., Gamra H., Kiwan G.S. (2019). Efficacy and Safety of Low-Dose Colchicine after Myocardial Infarction. N. Engl. J. Med..

[16] Feng Y., Ye D., Wang Z., Pan H., Lu X., Wang M., Xu Y., Yu J., Zhang J., Zhao M. (2022). The Role of Interleukin-6 Family Members in Cardiovascular Diseases. Front. Cardiovasc. Med..

[17] Bartoli-Leonard F., Zimmer J., Sonawane A.R., Perez K., Turner M.E., Kuraoka S., Pham T., Li F., Aikawa M., Singh S. (2023). NLRP3 Inflammasome Activation in Peripheral Arterial Disease. J. Am. Heart Assoc..

[18] ESC Guidelines on Peripheral Arterial Diseases (Diagnosis and Treatment of). https://www.escardio.org/Guidelines/Clinical-Practice-Guidelines/Peripheral-Artery-Diseases-Diagnosis-and-Treatment-of.

[19] Williams B., Mancia G., Spiering W., Agabiti Rosei E., Azizi M., Burnier M., Clement D.L., Coca A., de Simone G., Dominiczak A. (2018). 2018 ESC/ESH Guidelines for the Management of Arterial Hypertension. Eur. Heart J..

[20] Piepoli M.F., Hoes A.W., Agewall S., Albus C., Brotons C., Catapano A.L., Cooney M.-T., Corrà U., Cosyns B., Deaton C. (2016). 2016 European Guidelines on Cardiovascular Disease Prevention in Clinical Practice: The Sixth Joint Task Force of the European Society of Cardiology and Other Societies on Cardiovascular Disease Prevention in Clinical Practice (Constituted by Representatives of 10 Societies and by Invited Experts)Developed with the Special Contribution of the European Association for Cardiovascular Prevention & Rehabilitation (EACPR). Eur. Heart J..

[21] Visseren F.L.J., Mach F., Smulders Y.M., Carballo D., Koskinas K.C., Bäck M., Benetos A., Biffi A., Boavida J.-M., Capodanno D. (2021). 2021 ESC Guidelines on Cardiovascular Disease Prevention in Clinical Practice. Eur. Heart J..

[22] Wood D.M. (2005). “Pack Year” Smoking Histories: What about Patients Who Use Loose Tobacco?. Tob. Control.

[23] Lancellotti P., Zamorano J.L., Habib G., Badano L. (2016). The EACVI Textbook of Echocardiography.

[24] Erikson U. (1976). Technique of Coronary Angiography. Acta Radiol. Diagn..

[25] Omeh D.J., Shlofmitz E. (2023). Angiography. StatPearls.

[26] Wijnand J.G.J., Zarkowsky D., Wu B., van Haelst S.T.W., Vonken E.-J.P.A., Sorrentino T.A., Pallister Z., Chung J., Mills J.L., Teraa M. (2021). The Global Limb Anatomic Staging System (GLASS) for CLTI: Improving Inter-Observer Agreement. J. Clin. Med..

[27] Cerqueira L.d.O., Duarte E.G., Barros A.L.d.S., Cerqueira J.R., de Araújo W.J.B. (2020). WIfI Classification: The Society for Vascular Surgery Lower Extremity Threatened Limb Classification System, a Literature Review. J. Vasc. Bras..

[28] Alexander Y., Osto E., Schmidt-Trucksäss A., Shechter M., Trifunovic D., Duncker D.J., Aboyans V., Bäck M., Badimon L., Cosentino F. (2021). Endothelial Function in Cardiovascular Medicine: A Consensus Paper of the European Society of Cardiology Working Groups on Atherosclerosis and Vascular Biology, Aorta and Peripheral Vascular Diseases, Coronary Pathophysiology and Microcirculation, and Thrombosis. Cardiovasc. Res..

[29] Berenji Ardestani S., Eftedal I., Pedersen M., Jeppesen P.B., Nørregaard R., Matchkov V.V. (2020). Endothelial Dysfunction in Small Arteries and Early Signs of Atherosclerosis in ApoE Knockout Rats. Sci. Rep..

[30] Tunstall-Pedoe H., Peters S.A.E., Woodward M., Struthers A.D., Belch J.J.F. (2017). Twenty-Year Predictors of Peripheral Arterial Disease Compared with Coronary Heart Disease in the Scottish Heart Health Extended Cohort (SHHEC). JAHA.

[31] Saenz-Pipaon G., Martinez-Aguilar E., Orbe J., González Miqueo A., Fernandez-Alonso L., Paramo J.A., Roncal C. (2021). The Role of Circulating Biomarkers in Peripheral Arterial Disease. Int. J. Mol. Sci..

[32] Fukase T., Dohi T., Kato Y., Chikata Y., Takahashi N., Endo H., Doi S., Nishiyama H., Okai I., Iwata H. (2021). Long-Term Impact of High-Sensitivity C-Reactive Protein in Patients with Intermittent Claudication Due to Peripheral Artery Disease Following Endovascular Treatment. Heart Vessel..

[33] Demir K., Avci A., Altunkeser B.B., Yilmaz A., Keles F., Ersecgin A. (2014). The Relation between Neutrophil-to-Lymphocyte Ratio and Coronary Chronic Total Occlusions. BMC Cardiovasc. Disord..

[34] Kim S., Eliot M., Koestler D.C., Wu W.-C., Kelsey K.T. (2018). Association of Neutrophil-to-Lymphocyte Ratio with Mortality and Cardiovascular Disease in the Jackson Heart Study and Modification by the Duffy Antigen Variant. JAMA Cardiol..

[35] Seo I.-H., Lee Y.-J. (2022). Usefulness of Complete Blood Count (CBC) to Assess Cardiovascular and Metabolic Diseases in Clinical Settings: A Comprehensive Literature Review. Biomedicines.

[36] Angkananard T., Anothaisintawee T., McEvoy M., Attia J., Thakkinstian A. (2018). Neutrophil Lymphocyte Ratio and Cardiovascular Disease Risk: A Systematic Review and Meta-Analysis. Biomed. Res. Int..

[37] Taurino M., Aloisi F., Del Porto F., Nespola M., Dezi T., Pranteda C., Rizzo L., Sirignano P. (2021). Neutrophil-to-Lymphocyte Ratio Could Predict Outcome in Patients Presenting with Acute Limb Ischemia. J. Clin. Med..

[38] Coelho N.H., Coelho A., Augusto R., Semião C., Peixoto J., Fernandes L., Martins V., Canedo A., Gregório T. (2021). Pre-Operative Neutrophil to Lymphocyte Ratio Is Associated With 30 Day Death or Amputation After Revascularisation for Acute Limb Ischaemia. Eur. J. Vasc. Endovasc. Surg..

[39] Russu E., Mureșan A.V., Arbănași E.M., Kaller R., Hosu I., Voidăzan S., Arbănași E.M., Coșarcă C.M. (2022). The Predictive Role of NLR and PLR in Outcome and Patency of Lower Limb Revascularization in Patients with Femoropopliteal Disease. J. Clin. Med..

[40] Erturk M., Cakmak H.A., Surgit O., Celik O., Aksu H.U., Akgul O., Gurdogan M., Bulut U., Ozalp B., Akbay E. (2014). Predictive Value of Elevated Neutrophil to Lymphocyte Ratio for Long-Term Cardiovascular Mortality in Peripheral Arterial Occlusive Disease. J. Cardiol..

[41] Toor I.S., Jaumdally R.J., Moss M.S., Babu S.B. (2008). Preprocedural Neutrophil Count Predicts Outcome in Patients with Advanced Peripheral Vascular Disease Undergoing Percutaneous Transluminal Angioplasty. J. Vasc. Surg..

[42] Liang R.-F., Li M., Li J.-H., Zuo M.-R., Yang Y., Liu Y.-H. (2019). The Significance of Preoperative Hematological Inflammatory Markers in Patients with Meningiomas. Clin. Neurol. Neurosurg..

[43] Gary T., Pichler M., Belaj K., Hafner F., Gerger A., Froehlich H., Eller P., Rief P., Hackl G., Pilger E. (2013). Platelet-to-Lymphocyte Ratio: A Novel Marker for Critical Limb Ischemia in Peripheral Arterial Occlusive Disease Patients. PLoS ONE.

[44] Liu N., Sheng J., Pan T., Wang Y. (2019). Neutrophil to Lymphocyte Ratio and Platelet to Lymphocyte Ratio Are Associated with Lower Extremity Vascular Lesions in Chinese Patients with Type 2 Diabetes. Clin. Lab..

[45] Walzik D., Joisten N., Zacher J., Zimmer P. (2021). Transferring Clinically Established Immune Inflammation Markers into Exercise Physiology: Focus on Neutrophil-to-Lymphocyte Ratio, Platelet-to-Lymphocyte Ratio and Systemic Immune-Inflammation Index. Eur. J. Appl. Physiol..

[46] Stojkovic Lalosevic M., Pavlovic Markovic A., Stankovic S., Stojkovic M., Dimitrijevic I., Radoman Vujacic I., Lalic D., Milovanovic T., Dumic I., Krivokapic Z. (2019). Combined Diagnostic Efficacy of Neutrophil-to-Lymphocyte Ratio (NLR), Platelet-to-Lymphocyte Ratio (PLR), and Mean Platelet Volume (MPV) as Biomarkers of Systemic Inflammation in the Diagnosis of Colorectal Cancer. Dis. Markers.

[47] Uzun F., Erturk M., Cakmak H.A., Kalkan A.K., Akturk I.F., Yalcin A.A., Uygur B., Bulut U., Oz K. (2017). Usefulness of the Platelet-to-Lymphocyte Ratio in Predicting Long-Term Cardiovascular Mortality in Patients with Peripheral Arterial Occlusive Disease. Postępy Kardiol. Interwencyjnej.

[48] Santoro L., Ferraro P.M., Nesci A., D’Alessandro A., Macerola N., Forni F., Tartaglione R., Gasbarrini A., Santoliquido A. (2021). Neutrophil-to-Lymphocyte Ratio but Not Monocyte-to-HDL Cholesterol Ratio nor Platelet-to-Lymphocyte Ratio Correlates with Early Stages of Lower Extremity Arterial Disease: An Ultrasonographic Study. Eur. Rev. Med. Pharmacol. Sci..

[49] Guetl K., Raggam R.B., Muster V., Gressenberger P., Vujic J., Avian A., Hafner F., Wehrschuetz M., Brodmann M., Gary T. (2019). The White Blood Cell Count to Mean Platelet Volume Ratio for the Prediction of Chronic Limb-Threatening Ischemia in Lower Extremity Artery Disease. J. Clin. Med..

[50] Gary T., Pichler M., Belaj K., Hafner F., Gerger A., Froehlich H., Eller P., Pilger E., Brodmann M. (2013). Neutrophil-to-Lymphocyte Ratio and Its Association with Critical Limb Ischemia in PAOD Patients. PLoS ONE.

[51] Tamhane U.U., Aneja S., Montgomery D., Rogers E.-K., Eagle K.A., Gurm H.S. (2008). Association between Admission Neutrophil to Lymphocyte Ratio and Outcomes in Patients with Acute Coronary Syndrome. Am. J. Cardiol..

[52] Taşoğlu İ., Sert D., Colak N., Uzun A., Songur M., Ecevit A. (2014). Neutrophil-Lymphocyte Ratio and the Platelet-Lymphocyte Ratio Predict the Limb Survival in Critical Limb Ischemia. Clin. Appl. Thromb. Hemost..

[53] Arbel Y., Finkelstein A., Halkin A., Birati E.Y., Revivo M., Zuzut M., Shevach A., Berliner S., Herz I., Keren G. (2012). Neutrophil/Lymphocyte Ratio Is Related to the Severity of Coronary Artery Disease and Clinical Outcome in Patients Undergoing Angiography. Atherosclerosis.

[54] Yuan L., Li L., Yu T., Yang Z., Jiang T., Ma Q., Qi J., Shi Y., Zhao P. (2020). The Correlational Study about Neutrophil-to-Lymphocyte Ratio and Exercise Tolerance of Chronic Obstructive Pulmonary Disease Patients. Medicine.

[55] Liao Y.-H., Sung Y.-C., Chou C.-C., Chen C.-Y. (2016). Eight-Week Training Cessation Suppresses Physiological Stress but Rapidly Impairs Health Metabolic Profiles and Aerobic Capacity in Elite Taekwondo Athletes. PLoS ONE.

[56] Mills J. (2023). Infrainguinal Disease: Surgical Treatment. Rutherford’s Vascular Surgery.

